# Acoustofluidic assembly of primary tumor-derived organotypic cell clusters for rapid evaluation of cancer immunotherapy

**DOI:** 10.1186/s12951-023-01786-6

**Published:** 2023-02-04

**Authors:** Zhuhao Wu, Zheng Ao, Hongwei Cai, Xiang Li, Bin Chen, Honglei Tu, Yijie Wang, Rongze Olivia Lu, Mingxia Gu, Liang Cheng, Xin Lu, Feng Guo

**Affiliations:** 1 Department of Intelligent Systems Engineering, Indiana University, Bloomington, IN 47405 USA; 2 Computer Science Department, Indiana University, Bloomington, IN 47408 USA; 3 Department of Neurological Surgery, Brain Tumor Center, Helen Diller Family Comprehensive Cancer Center, University of California San Francisco, California, CA 94143 USA; 4 Center for Stem Cell and Organoid Medicine (CuSTOM), Division of Pulmonary Biology, Division of Developmental Biology, Cincinnati Children’s Hospital Medical Center, Cincinnati, OH 45229 USA; 5 University of Cincinnati School of Medicine, Cincinnati, OH 45229 USA; 6 Department of Pathology and Laboratory Medicine, Brown University Warren Alpert Medical School, Lifespan Academic Medical Center, and the Legorreta Cancer Center at Brown University, Providence, RI 02903 USA; 7 Department of Biological Sciences, Boler-Parseghian Center for Rare and Neglected Diseases, Harper Cancer Research Institute, University of Notre Dame, Notre Dame, IN 46556 USA; 8 Melvin and Bren Simon Cancer Center, Indiana University School of Medicine, Indianapolis, IN 46202 USA

**Keywords:** Microfluidics, Acoustofluidics, Cell clusters, Cancer models, Cancer immunotherapy

## Abstract

Cancer immunotherapy shows promising potential for treating breast cancer. While patients may have heterogeneous treatment responses for adjuvant therapy, it is challenging to predict an individual patient’s response to cancer immunotherapy. Here, we report primary tumor-derived organotypic cell clusters (POCCs) for rapid and reliable evaluation of cancer immunotherapy. By using a label-free, contactless, and highly biocompatible acoustofluidic method, hundreds of cell clusters could be assembled from patient primary breast tumor dissociation within 2 min. Through the incorporation of time-lapse living cell imaging, the POCCs could faithfully recapitulate the cancer-immune interaction dynamics as well as their response to checkpoint inhibitors. Superior to current tumor organoids that usually take more than two weeks to develop, the POCCs can be established and used for evaluation of cancer immunotherapy within 12 h. The POCCs can preserve the cell components from the primary tumor due to the short culture time. Moreover, the POCCs can be assembled with uniform fabricate size and cell composition and served as an open platform for manipulating cell composition and ratio under controlled treatment conditions with a short turnaround time. Thus, we provide a new method to identify potentially immunogenic breast tumors and test immunotherapy, promoting personalized cancer therapy.

**Supplementary Information:**

The online version contains supplementary material available at 10.1186/s12951-023-01786-6.

## Introduction

Immunotherapy has shown great potential in clinical cancer treatment [1–5]. In breast cancer, especially triple-negative breast cancer (TNBC), checkpoint inhibitors (CPi) showed a nearly 40% response rate in a neoadjuvant setting [6]. However, owing to the heterogeneity of tumor microenvironment (TME) as well as diverse mechanisms of immune evasion in TNBC, it remains a grand challenge to predict and stratify patients' responses to CPi [7–10]. Various factors, including immune cell infiltration, tumor mutational burden, neoantigen load, and presence of immune suppressive cell types: myeloid-derived suppressor cells (MDSC), cancer-associated fibroblasts (CAFs), and regulatory T cells (Treg) could all contribute to CPi response/resistance [11–15]. Thus, it is challenging to develop a "one-size-fits-all" assay to model, analyze, and predict TNBC responses to CPi in immunotherapy.

 To date, emerging technologies utilizing patient tumor-derived materials have shown promise to recapitulate TME and model responses to CPi ex vivo. Microfluidic co-cultures of immune cells and tumor fragments could model patient responses to CPi on a chip [16–18]. Additionally, the co-culture of patient-derived organoids and immune cells could also be utilized to study tumor immunity [7, 19, 20]. Finally, dissociated tumors could also be aggregated to study tumor-immune interactions under various treatments [21–23]. Although these models are promising, their adoption in evaluating immunotherapies is limited by three major factors. First, the models should be uniform in morphology and cell distribution for making the testing results more reliable [24–35]. Second, the cost-efficient, time-saving, and simple establishment of the models should be improved for the parallel interrogation of multiple treatments with fast turn-around results [36–50]. Last, the model should preserve the various immune components of the original tumor to recapitulate their functions and dynamic responses to CPi therapies [51–56].

To address the challenges in current tumor models, we develop patient breast tumor-derived organotypic cell clusters (POCCs) using a label-free, contact-free, and highly biocompatible acoustofluidic assembly method. The method could rapidly reconstruct cultures of original tumors’ TME for rapid evaluation of the immune responses to donor-matched T cells in breast tumors. Uniform POCCs can be acoustically assembled within 2 min and preserved all the cell components from the original tumors. Besides, as an open-operation platform, donor-matched T cells could be added directly to the cultures quantitatively assess the cytotoxicity of tumor-infiltrating T cells within a rapid turn-around time of 12 h under the treatment of CPi. We demonstrated that this method could be adopted to study the complex immune cell cross-talks in breast tumors and evaluate immunotherapy ex vivo in a reliable, rapid, and versatile manner.

## Results and discussion

### Formation of primary tumor-derived organotypic cell clusters (POCCs) using acoustofluidics

**Fig. 1:**
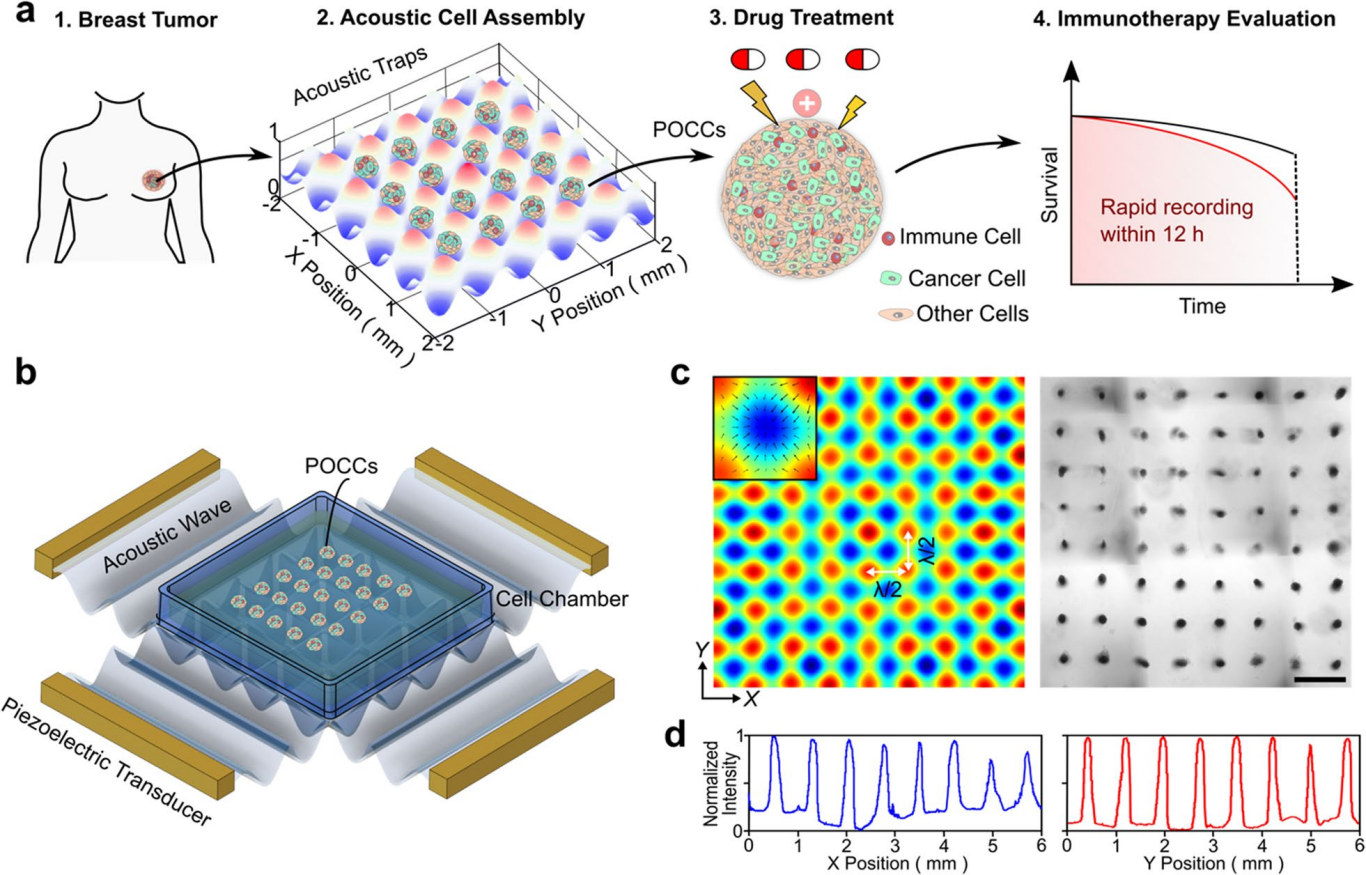
Acoustofluidic assembly of primary tumor-derived organotypic cell clusters (POCCs). **a** Schematic of evaluating immunotherapies using POCCs derived from breast cancer patients within 12 h. **b** Schematic of an acoustic cell assembly device. **c** The simulated acoustic pressure distribution and acoustically assembled 3D cell clusters in Matrigel. **d** The normalized intensity of cell clusters including the distance information in two directions. Scale bar: 1 mm

The acoustic cell assembly platform was developed for the rapid formation of TME-containing POCCs for personalized cancer therapy (Fig. 1a). This platform consists of four piezoelectric transducers (PZTs) and a fabricated cell chamber. After applying radio frequency (RF) signals, the acoustic cell assembly device can generate a uniform acoustic field (Fig. 1b), and push cells towards pressure nodes within 2 min of operation (Additional file 1: Fig. S1), thereby forming the patterning array of cell clusters (Fig. 1c). The distances between two adjacent cell clusters were approximately 750 μm in both $x$ and $y$ directions, showing uniformity of clusters’ size and distribution (Fig. 1d). Notably, our platform was biocompatible and versatile. On the one hand, this platform can fabricate cell clusters with customized sizes by tuning the cell concentration. Besides, high cell viability can be achieved using this method. E0771 tumor cell cluster growth was analyzed with the initial cell concentration set as 1.6 million/mL (Additional file 1: Fig. S2). From day 1 to day 4, the area of cell clusters increased from 4.6 × 10^4^ to 9 × 10^4^ μm^2^. Hundreds of uniform cell clusters in Matrigel could be assembled from single cell suspension of patient primary breast tumor dissociation. Compared with the other cell spheroid formation methods (e.g., hanging drop or U-bottom plates, which use gravity to form very loose cell clusters), our approach can rapidly form tight contacts of cells within 2 min, highlighting its potential in preserving tumor immune microenvironment and testing treatment response of an individual cancer patient.

### Preservation of tumor immune microenvironment

**Fig. 2:**
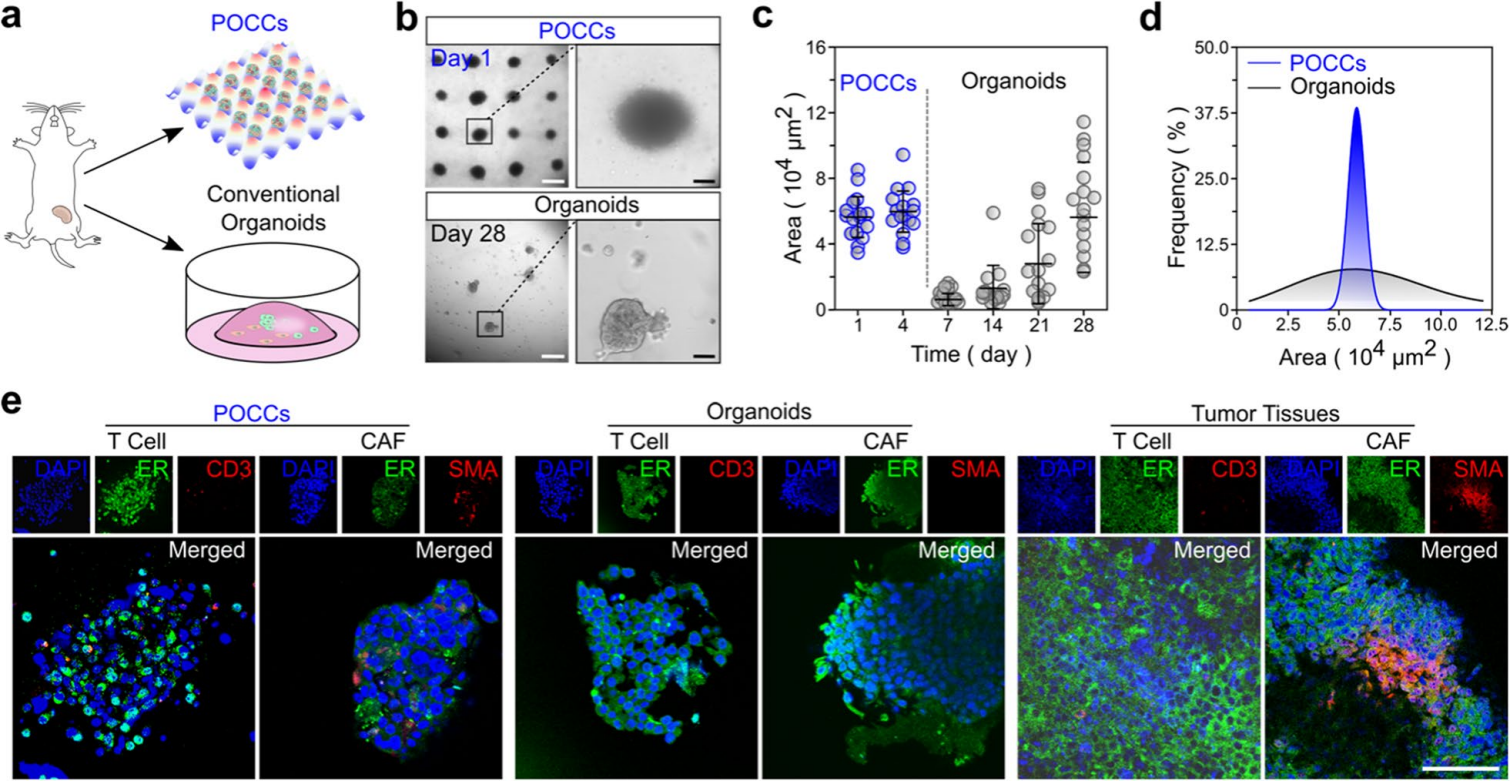
Formation of primary tumor-derived organotypic cell clusters (POCCs) derived from primary mouse tumors. **a** Schematics show the comparison of POCCs and conventional organoids from mouse orthotopic breast tumors, respectively. **b** Formation of POCCs (day 1) and Matrigel organoids (day 28) with similar size. White scale bar: 500 μm; Black scale bar: 100 μm. **c** Growth of POCCs and Matrigel organoids from day 1 to day 4 and day 1 to day 28, respectively. **d** Size distribution in POCCs on day 1 and Matrigel organoids on day 28. **e** Cell components in POCCs, Matrigel organoids, and primary tumor tissues. DAPI (nucleus), ER (tumor), SMA (CAF), CD3 (immunocyte). Scale bar: 100 μm

Our method enables the rapid generation of size-uniform 3D cultures or POCCs, which is superior to the conventional method. Conventional organoids needed 28 days to grow up to reach the same size (6 × 10^4^ μm^2^) as the POCCs on day one (Fig. 2a–c). Additionally, POCCs showed higher uniformity compared with conventional organoids (Fig. 2d). To test our hypothesis that POCCs could preserve the TME components, we used orthotopically implanted, syngeneic breast tumor model E0771 for acoustic assembly. We analyzed the cell components of POCCs including tumor cells (marker: estrogen receptor, ER), CAFs (marker: alpha-smooth muscle actin, aSMA), and T cells (marker: CD3) by immunofluorescence staining (Fig. 2e). We found the tumor cells, immune cells, and CAFs were preserved in POCCs (at day 2), which showed a high degree of similarity to the tumor tissue. In comparison, conventional organoids (at day 28) showed the presence of only tumor cells. Together, the results indicate that the TME components could be faithfully preserved in POCCs within a short culture time.

### Model tumor responses to cancer immunotherapy

**Fig. 3:**
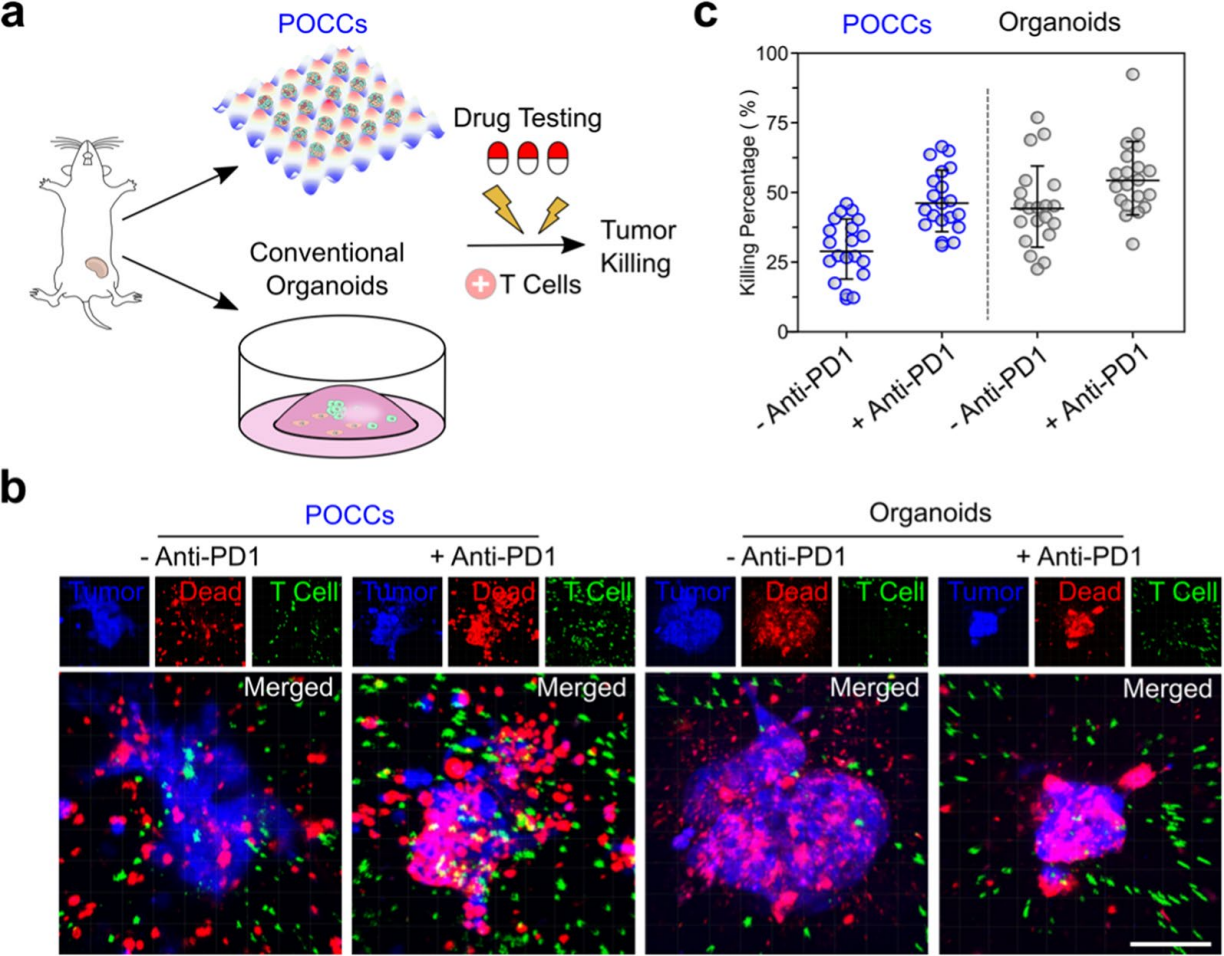
Modeling treatment response to immunotherapy using primary mouse samples. **a** Schematics of the working flow to validate cytotoxicity of CD8+ T cells in four groups including POCCs/T cells with or without anti-PD1, and Matrigel organoids/T cells with or without anti-PD1. **b** T cells’ killing results in four different groups. The red color represents the dead cells, the green color represents the T cells, and the blue color represents all the cells from POCCs or Matrigel organoids. **c** Statistical analysis of T cell’s killing efficiencies in four groups. Scale bar: 150 μm

To validate that the TME components captured in POCCs remain functionally active, and could respond to immune therapy, we compared T cell-mediated cytotoxicity in POCC co-cultures versus that with conventional organoids. We added OT-I T cells with transgenic OVA recognizing T cell receptor (TCR) to E0771-OVA orthotopic syngeneic tumor-derived POCCs and conventional organoids for 24 h and quantified the tumor cell death in the co-culture (Fig. 3a). First, we labeled POCCs and conventional organoids with cell tracker dye in blue color, T cells in green color, and then analyzed the dead cells with a red color cell death indicator (SYTOX red) (Fig. 3b). The data showed the POCCs were more resistant to OT-I mediated cell death likely due to the preservation of immune suppressive TME components (Fig. 3c). With anti-PD1 treatment, the cell death in POCCs was increased, indicating the functional recapitulation of CPi in our model. Taken together, the experimental results indicate that co-culture of T cells with POCCs could be utilized to replicate CPi efficacy ex vivo.

### Rapid evaluation of cancer immunotherapy using breast cancer patient tissues

**Fig. 4:**
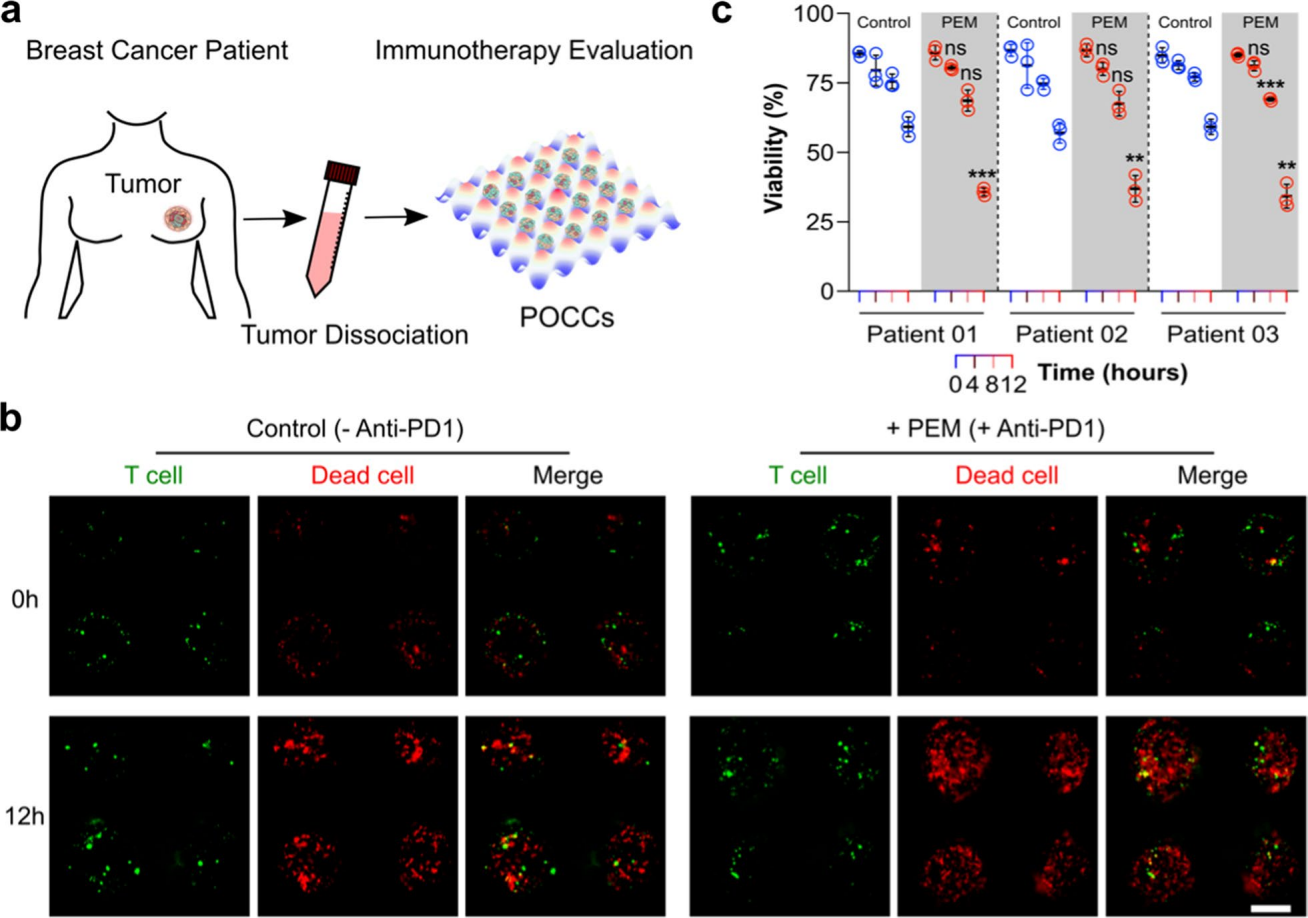
Evaluation of cancer immunotherapy using patient breast tumor tissues. **a** Coculture process of TILs and the dissociated tumor cells. **b** The typical killing results (left: control group; right: PEM-treated group) of T cells over 12 h along POCCs. The red color represents the dead cell, green color represents the T cell. **c** Statistical analysis of TIL’s killing efficiency in two groups among three breast cancer patients. *n* = 3 biological replicates, statistical analysis was performed using two-sample Student’s *t*-test, statistical significance was denoted as: ns p > 0.05, **p < 0.01, ***p < 0.005. Data are mean ± s.d. Scale bar: 200 μm

We next evaluated the application of POCCs in a breast cancer patient setting, within a clinically actionable 12-h time frame. POCCs were established from three surgically resected breast tumors and CD8+ TILs were isolated from the immunocyte-containing media from the specimen tube that the primary tumor was shipped in (Fig. 4a). CD8+ TILs were added for the formation of POCCs. The co-culture was then treated with a clinically approved anti-PD1 CPi (Pembrolizumab, PEM) with a concentration of 3 μg/mL. Images of tumor cell death in control and CPi-treated groups were captured at 0 h and 12 as shown in Fig. 4b. Tumor cells death was enhanced in CPi-treated groups in the individual quantitative data (Fig. 4c). Our data suggest that POCCs can be used to evaluate the functionality of TILs in breast cancer and could be implemented into precision immunotherapies in the clinic.

## Conclusion

Here, we developed acoustically assembled POCCs that could preserve all the TME components from original tumors, enabling the evaluation of treatment efficacy of immunotherapies with a short turn-around time (12 h). The POCCs could then be subject to T cell co-cultures with autologous or engineered T cells to evaluate cytotoxicity and responses to CPi. The POCCs hold the potential to be adapted for the predictive assessment of treatment outcomes in clinical immunotherapies. Additionally, the T cells in our current co-culture system could be replaced with genetically engineered TILs, CAR-T, or TCR-Ts, making them easily adaptable to be developed as an assay for target discovery and companion diagnostics for cancer cell therapies. The device could be further developed for clinical applications. First, the acoustofluidic device can be optimized and integrated into an all-in-one prototype, allowing the automatic operation of biological sample introduction and acoustic assembly for translating the use of POCCs outside the acoustofluidic bioengineering laboratory. Second, the medium and system for POCCs' culture could be improved for the long-term preservation of rare cells' viability and functionality. Besides, further studies would be required to build the scientific correlation of immunotherapy responses between POCCs and patients. Finally, focusing on the questions in the immune-oncology will extend the applications of POCCs in studying immune responses, and may foster immune drug screening and discovery.

## Materials and methods

### Design, fabrication, and operation of acoustofluidic cell assembly device

The a device was designed to have a PMMA matrix with a cell chamber and four transducer chambers integrated with four piezoelectric transducers. The cell chamber was designed to have a dimension of 40 mm × 40 mm × 3 mm, and the transducer chamber was designed to have a dimension of 50 mm × 20 mm × 10 mm. These two pairs of transducers were inserted into the transducer chambers with an orthogonal arrangement. Applying radio frequency signals generated by function generators (TGP3152, Aim TTi, UK) and amplified by power amplifiers (LZY-22+, Mini-circuit, USA), two transducer pairs were excited at 1.006 MHz and 0.996 MHz, respectively. The acoustofluidic devices were excited using the function generators and amplifiers as we reported before[57–61].

### Establishment of orthotopic breast cancer tumors

Primary tumor cells were isolated from the C57BL6 (purchased from Envigo) mouse’s breast tumor. All animal experiments and procedures are approved by Indiana University Bloomington Institutional Animal Care and Use Committee (BIAUC) under protocol #16-022-20. The primary tumor of the mouse was set up based on orthotopic injection. Briefly, we prepared E0771-OVA cell suspension containing 0.5 million/mL cells and 50% (v/v) Matrigel. Subsequently, 50 μL of cell suspension per mouse was implanted into the mammary gland via subcutaneous injection. Finally, 2–3 weeks after the implantation, the tumor tissue was harvested into 4 °C sterilized PBS buffer for the next dissociation process.

### Preparation of mouse-derived tumor Matrigel organoids

We established the tumor Matrigel organoid protocol, based on our previous protocols[48, 62–65]. Several mouse tumor tissues were minced into small pieces and digested in serum-free DMEM/F12 containing 1–2 mg/mL collagenase (Sigma, C9407) on a shaker at 37 °C for 1–2 h. The digested tissue suspension was sequentially sheared using a 5 mL flamed glass Pasteur pipette. Following the shearing process, strained the suspension over a 100-μm filter with DMEF/F12. After repeating several times, collected the pellet with centrifugation at 400×*g* for 5 min. 5 mL of red blood cell lysis buffer (ThermoFisher SCIENTIFIC) was added to the pellet. Shook it for 5 min at room temperature. Subsequently, 5 mL PBS was added and centrifuged at 400×*g* for 5 min. The pellet was suspended in the cold breast organoid culture medium with 50% (v/v) Matrigel (Corning) [66]. 40 μL of drops with cell suspension was added onto prewarmed 24-well culture plates (Corning) at 37 °C for 30 min. Upon completed gelation, 400 μL of organoid culture medium was added. The medium was changed every 4 days and organoids can be passaged every 2 weeks: organoids were sheared through a flamed glass pipette and digested in 2 mL of 0.25% Trypsin–EDTA (Gibco). Organoid fragments were centrifuged and resuspended at a ratio (1:6) in cold Matrigel and cold organoid culture medium. Then, 40 μL of drops with cell suspension was added onto 24-well culture plates. Finally, 400 μL of organoid culture medium was added to each well for further culture.

### Isolation of mouse OT-I CD8+ cells

OT-I (C57BL/6-Tg(TcraTcrb)1100Mjb) mice have transgenic inserts for mouse Tcra-V2 and Tcrb-V5 genes, enabling the targeting behavior of OT-I CD8+ T cell to E0771-OVA tumor cells. OT-I CD8+ T cells were isolated from OT-I mouse spleen by using the Naïve CD8 T cell isolation kit (No. 130-096-543, Miltenyi Biotec.). Once the spleen was separated from the mouse, immediately milled into small pieces using the syringe, and filtered by a 30-μm filter. Then the small spleen pieces were centrifuged at 300×*g* for 7 min and collected by removing the supernatant in the colonial tube. 5 mL of ACK Lysing buffer was added into the colonial tube to remove the red blood cells. The suspension was pipetted up and down 10 times using 1 mL of the pipette and shaken for 5 min by hand at room temperature. 5 mL of PBS solution was then added to the colonial tube. The suspension was centrifuged at 300×*g* for 10 min. Then, the cell pellet was resuspended in 400 μL of Buffer per 10^8^ total cells. 100 μL of Naive CD8+ T Cell Biotin-Antibody Cocktail was added per 10^8^ total cells and mixed well and incubated for 5 min in a refrigerator (2–8 °C). Cells were washed by adding 10 mL of buffer per 10^8^ cells and centrifuged at 300×*g* for 10 min. The supernatant was removed, and the cells were resuspended in 500 μL of buffer per 10^8^ cells. The cell suspension was applied to the column with a magnetic field. The flow-through containing unlabeled cells was collected, representing the enriched naive CD8+ T cell fraction.

### Preparation of patient-derived breast tumor cells

The collection of breast cancer tissue from patients for the generation of tumor cultures has been performed according to the protocol approved by Indiana University Institutional Review Board (IRB # 1907977109). By following the tumor dissociation kit–human (Miltenyi Biotec Inc.), we got a high yield of tumor cells, stromal cells, and tumor-infiltrating lymphocytes, while preserving cell surface epitopes. The protocol began with removing fat, fibrous and necrotic areas from tumor tissue, then cut into small pieces of 2–4 mm, and transferring into gentle MACS C Tube containing the enzyme mix. Subsequently, these tissue pieces were dissociated into cell suspension on the gentleMACS Dissociator. Finally, the cell suspension was collected and processed immediately for downstream applications.

### Isolation of human CD8+ T cell from TILs

Human CD8+ T cells were isolated from the supernatant of the patient specimen by using the human CD8+ T cell isolation kit (No. 130-096-244, Miltenyi Biotec.). First, we prepared a solution containing PBS (PH 7.2), 0.5% bovine serum albumin (BSA), and 2 mM EDTA by diluting MACS BSA Stock Solution (#130-091-376) 1:20 with autoMACS Rinsing Solution (#130-091-222) as the buffer. The buffer was degassed before use. Upon arrival, the patient specimen was washed in a fresh RPMI medium. The supernatant was collected into 50 mL of the tube. Cell concentration was calculated by cell counter and collected by centrifuging at 300×*g* for 5 min. The cell pellet was resuspended in 40 μL of buffer per 10^7^ total cells. The suspension was added with 10 μL of CD8+ T cell Biotin-Antibody Cocktail per 10^7^ total cells and mixed well. Then, the cell suspension was incubated for 5 min in the refrigerator (4 °C). After 5 min, the suspension was added with 30 μL of buffer per 10^7^ total cells and 20 μL of CD8 + T cell Microbeads Cocktail per 10^7^ total cells. The cell suspension was incubated for 10 min in the refrigerator (4 °C). Cell magnetic separation was subsequently operated. LS column was placed in the magnetic field of a suitable MACS Separator. The column was rinsed with 3 mL of buffer. The cell suspension was applied to the column. The unlabeled cells passed through the column and represented the CD8+ T cells. The column was washed with 3 mL of buffer again to efficiently collect the unlabeled CD8+ T cells. The number of CD8+ T cells was calculated by the cell counter before use.

### Acoustic assembly of POCCs and isolation of CD8+ T cells

The dissociated cells from the tumor specimen and T cells were assembled into uniform cell clusters to investigate the killing performance of T cells and the function of the immune checkpoint inhibitor. CD8+ T cells were stained with cell tracker dye (DIO, V22886, ThermoFisher), and the dissociated cells from the tumor were mixed with dead cell dye (EthD-1, E1169, ThermoFisher) before the acoustic assembly process. Then, the stained CD8+ T cells and dissociated cells were mixed at a 1:10 ratio with a T cell culture medium. The cell concentration in the final was about 2 million/mL. Immediately, the mixed cell suspension was operated following the step of acoustic cell assembly. After the acoustic assembly, the co-cultures were cultured inside a small incubator on the microscope and automatically monitored for 12 h.

### Immunohistochemistry of POCCs and tumor tissues

The received tumor tissue and acoustic cell clusters were fixed in 4% paraformaldehyde followed by dehydration, paraffin embedding, sectioning, and standard H&E staining. Immunofluorescence was performed according to a previously published protocol. Briefly, POCCs were washed in PBS twice and blocked in 500 μL of blocking buffer for 60 min. The primary antibodies for EpCAM, CD3, and SMA were diluted at the ratio of 1:200 in the antibody dilution buffer. At the end of the blocking procedure, the blocking solution was aspirated completely. Acoustic cell clusters were incubated with primary antibodies at 4 °C overnight and washed 3 times with PBS solution. Secondary antibodies, Donkey anti-Rabbit IgG-Alexa 594 (Thermo Fisher, R37119), Donkey anti-Rat IgG-Alexa 488 (Thermo Fisher, A-21208), were diluted at a ratio of 1:500 in the antibody dilution buffer and samples were incubated with secondary antibodies for 2 h at room temperature in the dark. Then the samples were rinsed three times in PBS solution for 5 min each time. The acoustic cell clusters were immersed in a fructose-glycerol clearing solution and covered with coverslips. Stained samples were imaged using an X83 microscope.

## Supplementary Information

**Additional file 1: Figure. S1** Process of acoustic cell clustering and analysis of cell viability. **a** Acoustic cell assembly after 2 min. **b** Analysis of cell viability before and after acoustic signals. Scale bar: 0.5 mm. **Figure. S2** Growth of acoustically-assembled cell clusters. **a** The typical images of E0771 tumor cell clusters on day 1 and day 4. **b** Statistical analysis of the growth of cell clusters from day 1 to day 4. Scale bar: 250 μm.

## Data Availability

The dataset supporting the conclusions of this article is included within the article and its additional materials.
