# Supplementary material for: Acoustofluidic assembly of primary tumor-derived organotypic cell clusters for rapid evaluation of cancer immunotherapy
Source: J Nanobiotechnology. 2023 Feb 4;21:40. doi: 10.1186/s12951-023-01786-6 (PMC9899402; doi:10.1186/s12951-023-01786-6)
Supplement: Supplementary file 1 — Additional file 1: Figure. S1 Process of acoustic cell clustering and analysis of cell viability. a Acoustic cell assembly after 2 min. b Analysis of cell viability before and after acoustic signals. Scale bar: 0.5 mm. Figure. S2 Growth of acoustically-assembled cell clusters. a The typical images of E0771 tumor cell clusters on day 1 and day 4. b Statistical analysis of the growth of cell clusters from day 1 to day 4. Scale bar: 250 μm. [file 12951_2023_1786_MOESM1_ESM.pdf]

Supplementary Information

Acoustofluidic assembly of primary tumor-derived organotypic cell clusters for rapid evaluation of cancer immunotherapy

Zhuhao Wu,[1] Zheng Ao,[1*] Hongwei Cai,[1] Xiang Li, [1] Bin Chen,[1] Honglei Tu,[1] Yijie Wang,[2] Rongze Olivia Lu,[3] Mingxia Gu,[4,5] Liang Cheng,[6] Xin Lu,[7,8] and Feng Guo,[1,8*]

1. Department of Intelligent Systems Engineering, Indiana University, Bloomington, IN 47405, United States
2. Computer Science Department, Indiana University, Bloomington, IN 47408, United States
3. Department of Neurological Surgery, Brain Tumor Center, Helen Diller Family Comprehensive Cancer Center, University of California San Francisco, California, CA 94143, United States
4. Center for Stem Cell and Organoid Medicine (CuSTOM), Division of Pulmonary Biology, Division of Developmental Biology, Cincinnati Children's Hospital Medical Center, Cincinnati, OH 45229, United States
5. University of Cincinnati School of Medicine, Cincinnati, OH 45229, United States
6. Department of Pathology and Laboratory Medicine, Brown University Warren Alpert Medical School, Lifespan Academic Medical Center, and the Legorreta Cancer Center at Brown University, Providence, RI 02903, United States
7. Department of Biological Sciences, Boler-Parseghian Center for Rare and Neglected Diseases, Harper Cancer Research Institute, University of Notre Dame, Notre Dame, IN 46556, United States
8. Melvin and Bren Simon Cancer Center, Indiana University School of Medicine, Indianapolis, IN 46202, United States

*Corresponding email: aozheng.89@gmail.com and fengguo@iu.edu

Supplementary Figures

- **Figure S1 Process of acoustic cell assembly and analysis of cell viability**
- **Figure S2 The growth of acoustic cell clusters**

Supplementary Figures

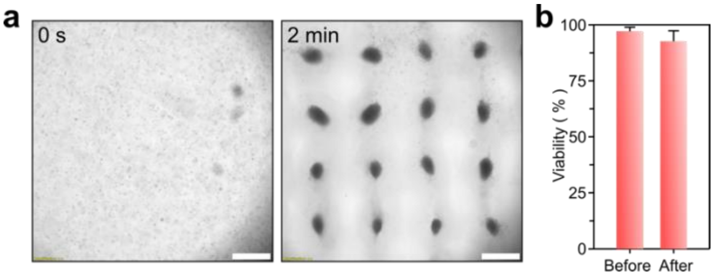

Figure. S1 Process of acoustic cell clustering and analysis of cell viability. (a) Acoustic cell assembly after 2 min. **(b)** Analysis of cell viability before and after acoustic signals. Scale bar: 0.5 mm.

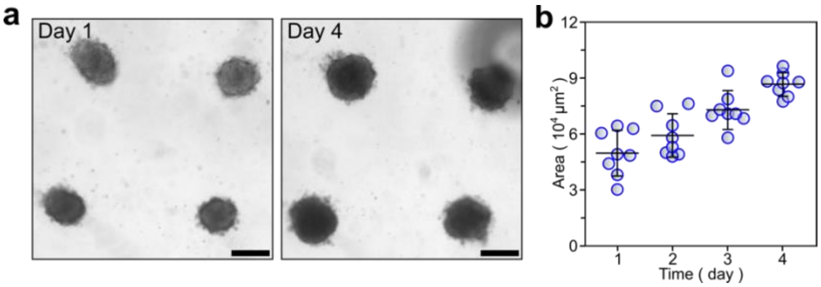

Figure. S2 Growth of acoustically-assembled cell clusters. (a) The typical images of E0771 tumor cell clusters on day 1 and day 4. **(b)** Statistical analysis of the growth of cell clusters from day 1 to day 4. Scale bar: 250 μm.
